# Uncertainty - a perennial

**DOI:** 10.1093/jhps/hnac028

**Published:** 2022-06-27

**Authors:** Richard E Field

**Affiliations:** Editor-in-chief - JHPS

2000 years ago, Pliny the Elder wrote that *The only certainty is that nothing is certain* [1].

In the ancient world, the closest thing to certainty was the rising and setting of the sun. Its timing may have been inconsistent, but the sun was sufficiently predictable that mankind deified it for many millennia. In the absence of certainty, humans form opinions. These are manifested in differences in habit, practice, debate and, sometimes, bloodshed.

Surgeons have debated the best approach to the hip for the last 150 years, and there is still no consensus. Hip replacement surgeons cannot say, with certainty, which method of implant fixation or which articular couple is best. Hip preservation surgeons debate when a joint should be realigned, when it should be recontoured and when it should be left untouched. We debate whether arthroscopies should be undertaken with patients lying on their side or their back. New debates emerge and our focus shifts. Many of us are now considering whether damaged articular cartilage can be made to regenerate and whether the surfaces of the joint are best separated applying traction with, or without, a peroneal post.

Over the centuries, advances in science have reshaped human perception, replaced gods with mathematical formulas and provided new ‘certainties’. Some surgeons hone their practice by emulating their teachers, trial and error and accumulated experience. Others try to evaluate their work through audit and research, using the study design models and mathematical analyses that are currently in vogue. Journals disseminate opinions, experience and scientific evaluation so that options can be debated and perhaps, a consensus may be achieved.

Hip preservation surgery is a relatively new field of surgical endeavour, and *JHPS* provides a forum for us to share our experiences, debate our uncertainties and report our findings. Medical and surgical interventions can be expensive, and healthcare funders are charged to ensure that finite resources are allocated in the most beneficial and cost-effective manner [2]. Femoroacetabular impingement affects individuals at a stage in their lives when they should be contributing tax to public service funds. The long-term sequelae of femoroacetabular impingement may precipitate joint degeneration [3] and lead to further healthcare expenditure in later life [4].

In issue 9.2, we have a paper from O’Donnell and his co-workers who have undertaken a systematic review and meta-analysis of randomised controlled trials, comparing arthroscopic hip surgery with targeted physiotherapy programmes for the treatment of femoroacetabular impingement syndrome [5]. This adds to the growing body of evidence [6–9] that healthcare funders can turn to when assessing where to allocate their resources and I look forward to receiving manuscripts from other investigators to better understand the health economics of available treatment options.

Another uncertainty that we encounter in our clinical practice is to identify what is causing hip impingement or instability. This is addressed by Dr Lerch and his co-investigators with their meticulous study on the interplay of femoral and acetabular versions [10]. This team has been investigating hip geometry for some years [11–13], and their latest paper provides valuable new insights into the interplay of femur and acetabulum.

A source of uncertainty facing any surgeon operating on skeletally immature patients is the concern that disturbance to open growth plates may have adverse, long-term consequences. Dr Sleth and her co-investigators’ paper investigating whether the capital femoral epiphysis grows after screw fixation for slipped capital femoral epiphysis [14] provides us with information to share with anxious parents. It allows us to tell parents that, while fixing a slipped capital epiphyses will have consequences on femoral neck angulation, it should not arrest proximal femoral growth.

I hope that you share my enjoyment of all the excellent papers in *JHPS* issue 9.2 and that *JHPS* will continue to help you unravel some of the uncertainties that face us every day.
